# Lessons about the reliability of congenital syphilis and vertical HIV transmission data learned from case reviews in Uruguay: a cross-sectional study

**DOI:** 10.1186/s12884-019-2516-z

**Published:** 2019-11-04

**Authors:** Susana Cabrera, Mariangela Freitas Silveira, Ana Visconti, Fabian García, Rafael Aguirre, Rodolfo Gomez Ponce de Leon, Jorge Quian, Suzanne J. Serruya

**Affiliations:** 1 STI-HIV/AIDS Program Area, Public Health Ministry, Montevideo, Uruguay; 2 Latin American Center of Perinatology, Women and Reproductive Health, Montevideo, Uruguay; 3 Sexual and Reproductive Health Program Area, Public Health Ministry, Montevideo, Uruguay; 4 Women’s Health Program Area, Public Health Ministry, Montevideo, Uruguay; 5 Public Health Ministry, Montevideo, Uruguay

**Keywords:** Syphilis, Congenital, HIV, Vertical transmission

## Abstract

**Background:**

In Uruguay it is mandatory to review all cases of positive HIV or reactive syphilis tests in pregnancy and peripartum. We compared the rates of mother-to-child transmission of syphilis and HIV detected by case reviews to those obtained from the usual surveillance system and described the characteristic of vertical transmission cases.

**Methods:**

This is a cross-sectional study performed with secondary data obtained from official government sources, for all the country cases of maternal to child transmission of HIV and syphilis from 2012 to 2017, with descriptive analyses. For congenital syphilis analyses, the following pregnancy characteristics were investigated: number of antenatal checks, gestational age at pregnancy diagnosis, gestational age at syphilis test and diagnosis, adequate treatment, and treatment of partners. Sociodemographic characteristics included type of health care (public/private), maternal age, distribution of ethnic minorities, maximum educational attainment, presence of partner, planned pregnancy, drug and alcohol use, domestic violence, previous maternal diagnosis of syphilis, and previous children with congenital syphilis.

**Results:**

Coverage of syphilis case reviews increased from 82% in 2014 to 97.4% in 2017. For HIV, this coverage reached 100% in 2017 and elimination of mother to child transmission was achieved. A marked decline in congenital syphilis was noted in the public health care sector, especially in the capital Montevideo, whereas the private sector has remained below the elimination target. Variables related with congenital syphilis in exposed children were late pregnancy diagnosis, < 5 antenatal checks, delayed diagnosis of gestational syphilis, lower rate of correct treatment for gestational syphilis, untreated partner, low maternal schooling, unplanned pregnancy, history of syphilis, and having other children with syphilis.

**Conclusion:**

The use of case reviews provided knowledge regarding the accurate number of mother-to-child transmission cases and the evolution of elimination of mother to child transmission in the country. The results suggest that rates must be adjusted, providing an opportunity to improve the reliability of surveillance data, and point the need to address specific gaps in order to improve the quality of care during pregnancy, delivery, and the neonatal period.

## Key messages

- Case reviews of congenital syphilis in Uruguay have produced better knowledge regarding the accurate number of cases.
- Case reviews allow assessments of EMTCT status.
- Surveillance rates must be adjusted according to case reviews.
- Several prevention strategies could be implemented at the primary care level to improve care and reduce congenital syphilis.

## Background

The global community has set the elimination of mother-to-child transmission (EMTCT) of HIV and syphilis as a public health priority. In 2007, an initiative for the EMTCT of HIV and syphilis led by the World Health Organization (WHO) was launched, based on a foundation of quality maternal and child health services, as advocated by the Millennium Development Goals (MDGs), and aimed at reducing child mortality and improving maternal health [1–3]. Those goals were also supported by the Pan American Health Organization (PAHO) since 2010 with the Plan of Action for Elimination of Mother-to-Child Transmission of HIV and Congenital Syphilis [4].

In any country, specific strategies for dual EMTCT of HIV and syphilis are influenced by local characteristics, including HIV and syphilis epidemiology, service delivery models, coverage of health services, and available resources [1].

With the aim of advancing toward universal coverage, a health system reform was begun in Uruguay in 2005 with the establishment of an Integrated National Health Care System (*Sistema Nacional Integrado de Salud*, SNIS) and creation of the National Health Insurance (*Seguro Nacional de Salud,* SNS). The SNS translated into increased population access to public health care services. The national health care policy, including health surveillance and inspection activities, is managed by the Ministry of Public Health [5]. Regarding mother/child health, 99% of deliveries in Uruguay are institutional and attended by skilled health professionals. The national health care policy emphasizes early antenatal care (starting at < 12 weeks of gestational age) as well as an increase in the number of antenatal checks. According to the Perinatal Information System (*Sistema Informático Perinatal,* SIP), the rate of early antenatal care was 78% in 2016, with 93% of pregnant women having more than 5 antenatal checks [5].

Elimination of vertical syphilis and HIV transmission is among the 15 critical problems to be prioritized as part of the Ministry of Public Health’s strategic plan for 2020 [6]. Integration of sexually transmitted infections (STI) and HIV prevention into the country’s sexual and reproductive health policy should be a special highlight of the EMTCT strategy in Uruguay. In 1997, syphilis and HIV testing during pregnancy became mandatory in the country, along with the provision of antiretroviral therapy (ART) and feeding formulas for infected mothers [6]. Since 2002, rapid syphilis and HIV tests have been routinely performed in the country’s birth centers. Rapid testing was extended to the primary care level in 2013 and penicillin is available at the clinics after the first positive test both for the pregnant women and their partners. In that same year, a third syphilis and HIV screening was introduced, so that screening is now performed at the first antenatal check and during the second and third pregnancy trimesters [6]. Since 2014, the presence of sexual partners in antenatal visits at least once in the first and third trimesters has been encouraged, with advice to perform syphilis testing in accordance with the national recommendations for EMTCT of syphilis and HIV [7].

A crucial measure instituted along this period (in 2013) was the mandatory reporting and review of cases with positive HIV tests or reactive syphilis tests during the pregnancy and peripartum, with incorporation of these reviews as production targets (i.e., part of a performance-based incentive scheme) for all SNIS institutions [8]. Individual congenital syphilis case reviews are recommended by WHO to evaluate the use of consistent and correct case definitions, as well to detect under or overreporting [1]. Since 2017, a new production target, named “Boy, Girl, and Woman,” focusing on pregnancy and on the monitoring of children until 3 years of age, introduced mandatory offer of VDRL/RPR and HIV testing for women and their partners across the SNIS [https://www.paho.org/uru/index.php?option=com_docman&view=document&slug=ordenanza-n-1119-2018-msp-etmi-plus&layout=default&alias=606-ordenanza-n-1119-2018-msp-etmi-plus&category_slug=publications&Itemid=307 ]. This target serves as an incentive to the review of cases by health care institutions.

Given this scenario, the objective of the present work was to compare the rates of mother-to-child transmission of syphilis and HIV detected by case reviews to those obtained from the usual surveillance system. Also, case reviews were examined to identify gaps in care processes and variables associated with vertical transmission, generating information that can be useful for training and the implementation of corrective actions.

## Methods

This is a cross-sectional study performed with secondary data obtained from public government sources: electronic live birth certificate (LBC, which provides information on syphilis and HIV test results), SIP, DST-HIV-AIDS Programs, epidemiological data on HIV/AIDS in Uruguay (for the years 2012–2017), vital statistics, and congenital syphilis and HIV case review database. Uruguay is a South American country with a population of 3,480,222, with 95% living in urban areas [9]. The country has a high human development index (HDI), of 0.795 [10], and was classified by the World Bank as a high income country since 2013 [11]. All the congenital syphilis and Maternal to child transmission of HIV cases from 2014 to 2017 were included. Congenital syphilis cases were defined using the WHO surveillance definition: a live birth or fetal death at > 20 weeks of gestation or > 500 g (including stillbirths) born to a woman with positive syphilis serology and without adequate syphilis treatment, or a live birth, stillbirth or child aged < 2 years born to a woman with positive syphilis serology or with unknown serostatus, and with laboratory and/or radiographic and/or clinical evidence of syphilis infection (regardless of the timing or adequacy of maternal treatment) [1]. For the vertical transmission of HIV, diagnosis was confirmed by nucleic acid testing.

Congenital syphilis analyses also included a description of the following pregnancy characteristics: women with < 5 antenatal checks, mean gestational age at pregnancy diagnosis in weeks, pregnancy diagnosis at > 14 weeks, mean gestational age at first syphilis diagnosis in pregnancy, first syphilis test at > 14 weeks, mean gestational age at the diagnosis of syphilis during pregnancy, diagnosis of syphilis at delivery or puerperium, adequate treatment, and treatment of partners (yes or no).

Sociodemographic characteristics were also described: type of health care (public/private), mothers with age < 19 years, distribution of ethnic minorities, maximum educational attainment (illiterate, incomplete elementary, complete elementary, incomplete high school, complete high school, university level), living with partner, planned pregnancy, drug and alcohol use, domestic violence, previous maternal diagnosis of syphilis, and previous children with congenital syphilis.

Rates of congenital syphilis and vertical transmission of HIV were described. Antenatal care and sociodemographic characteristics (all variables collected at the audits), as means or percentages, are described according to the congenital syphilis outcome. Characteristics of vertical transmission of HIV cases were described individually.

Information for the calculation of EMTCT indicators for HIV and congenital syphilis is obtained from the bureau for epidemiological surveillance (DEVISA) and the SIP, mandatorily used by all public and private pregnancy care services in Uruguay. Birth center databases provide aggregate national data or specific data sets for different provinces, non-metropolitan areas, or by health care sector (public or private).

### Case reviews

Two Ministry of Health Program Areas (STI-HIV/AIDS and Sexual and Reproductive Health) are in charge of analyzing case review data and of producing reports regarding process and performance indicators. The following actions have been developed by the Program Areas in association with the case reviews: design of case review form, validation of case review form [12], design of an online tool for submission of syphilis case reviews, and inclusion of information regarding completed syphilis and HIV case reviews as part of the production targets. Case reviews are triggered by the identification of a “reactive” syphilis or HIV test in women during the pregnancy, labor, or puerperium, or in neonates.

The syphilis case review form must be filled for all women with a reactive syphilis test (non-treponemal or rapid treponemal test) during pregnancy, labor, or puerperium, and for all newborns with a reactive non-treponemal cord blood test. HIV case review forms are completed for all women with HIV diagnosis who become pregnant, or for those diagnosed during pregnancy, labor, or early puerperium. HIV-exposed children are followed until HIV infection is confirmed or ruled out. The mothers are also followed-up regarding adherence to ART.

The consolidated SIP database and LBC data are examined in search of “reactive” syphilis and HIV tests. This information is contrasted to the case reviews submitted by health care institution. This triangulation allows the construction of a case review database, with identification of missing cases (not spontaneously submitted), which can then be requested. For the case reviews, specific forms are filled and submitted by health care providers via e-mail to the STI-HIV/AIDS Program Area. In 2017, an online tool was created for syphilis case reviews, with assignment of a username to each health care provider.

The technical department in charge of delivery/C-section or abortion care at each institution performs the case review. The form must include additional data from the clinical history and the SIP. The institution where the final pregnancy care was provided is in charge of submitting to the Ministry of Health the completed case review form including a copy of SIP history no later than 45 days following the birth or stillbirth.

By analyzing these records, the following information can be obtained: socio-demographic information, information regarding current pregnancy (gestational age at first antenatal check, number of checks), information provided at syphilis or HIV testing (gestational age at first test and at first test with reactive result, results of non-treponemal tests expressed as a titer, results of follow-up, types of tests performed), information regarding treatment, and data about the partner, the delivery, and the newborn. For the present study, the analysis of years 2015–2017 included complementary information obtained from SIP regarding ethnicity, education, use of alcohol and drugs, violence, and having a stable partner.

## Results

Coverage of syphilis case reviews (submission of the form in relation to the total number of reactive syphilis tests) increased from 82% in 2014 to 95.3% in 2015, 97.3% in 2016 and 97.4% in 2017. The coverage of HIV case reviews was 97% in 2014, 98% in 2015 and 100% in 2016 and 2017. For HIV, it should be noted that, even in the absence of a case review, 100% of the exposed children are followed-up.

Table 1 shows the results of EMTCT impact indicators for congenital syphilis using as data source the routine reporting system (SIP) vs. case reviews, as well as data related to vertical HIV transmission obtained from case reviews performed from 2012 to 2017. The data shows that, for HIV, the vertical transmission rate reached the EMTCT goal of < 2% in the 2 final years of analysis.

**Table 1:** Annual rate of congenital syphilis cases and incidence of vertical HIV transmission reported per 1000 live births, based on case review data, Uruguay, 2012–2017

Data source	Year					
	2012	2013	2014	2015	2016	2017
CS rate - routine reporting system (SIP)	–	1.7/1000	1.2/1000	1.1/1000	1.0/1000	0.8/1000
CS rate - case reviews	–	2.0/1000	2.3/1000	1.8/1000	1.5/1000	1.1/1000
No. births	48,059	48,681	48,368	48,915	47,058	43,036
No. exposed children (born from HIV-infected mothers)	142	124	136	112	130	102
No. children infected	9	2	4	2	2	2
Vertical transmission rate	6.3	1.6	2.9	1.8	1.5	1.9
HIV transmission rate	0.19	0.04	0.08	0.02	0.04	0.05

*CS* congenital syphilis

The congenital syphilis rate obtained from case reviews was consistently higher than that obtained from the usual system. This indicates that some reactive tests included for case review were not reported to the surveillance system as cases of congenital syphilis.

**Fig. 1:**
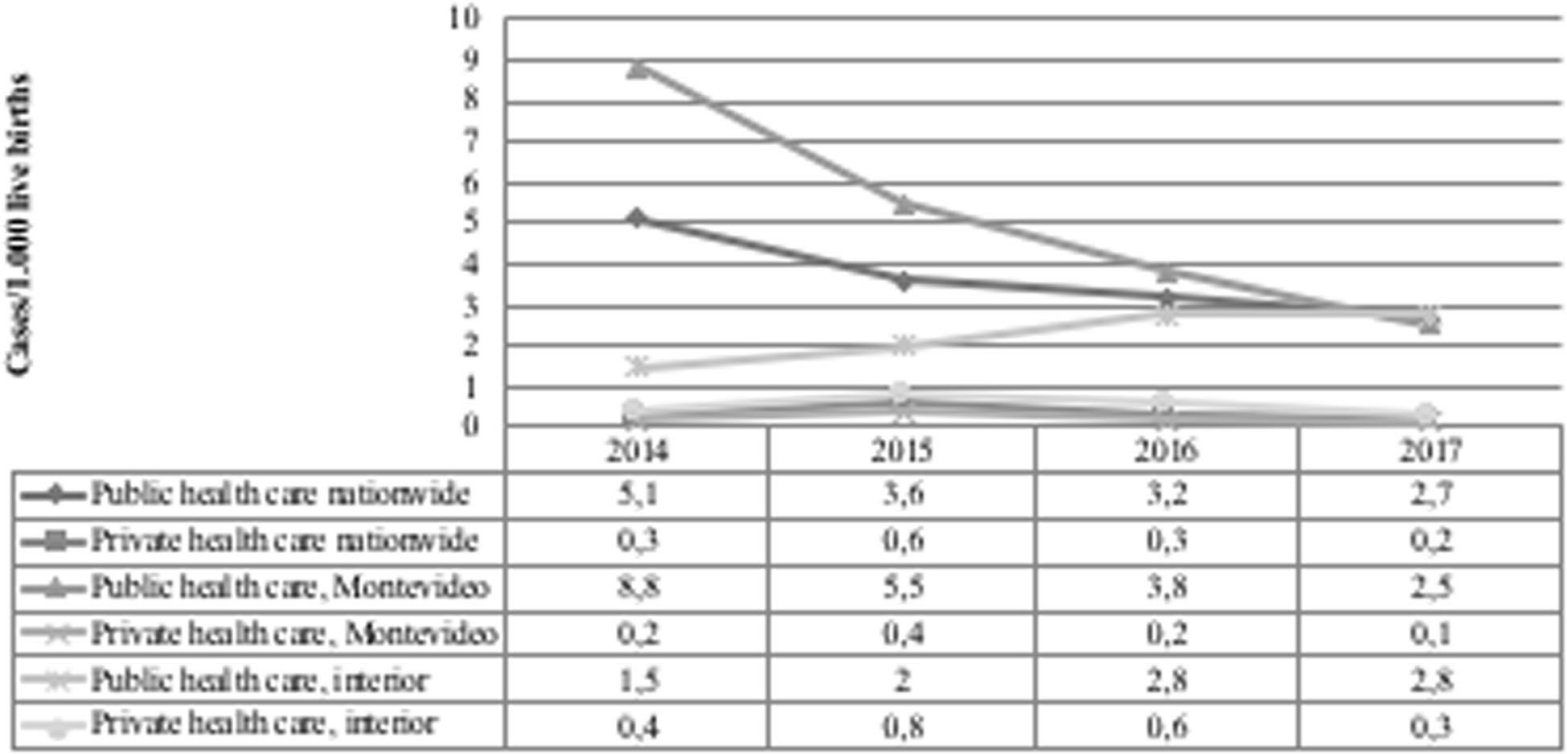
Evolution of congenital syphilis infection rate according to health care sector and geographic location in Uruguay, 2014–2017

Regarding congenital syphilis, Fig. 1 shows a marked decrease in the public health care sector, resulting in a positive impact on the decline observed at the national level. In turn, the private sector has remained below or close to the elimination rate of 0.5%. The reduction in the national rate of congenital syphilis associated with the public sector resulted from a major decline recorded in the capital city of Montevideo. Conversely, an increase was observed in the country interior. For the private sector, a decline was detected in the last year of the series for both the overall national rate and Montevideo, with achievement of the elimination target including the country interior. (Fig. 1).

Table 2 shows a description of pregnancy characteristics for congenital syphilis cases after a diagnosis in pregnancy in 2014, 2015, 2016 and 2017. We can observe a higher prevalence of delayed pregnancy diagnosis, fewer than 5 antenatal checks, delayed syphilis diagnosis or diagnosis of gestational syphilis at delivery or puerperium, lower rate of correct treatment of congenital syphilis, and untreated partner.

**Table 2:** Pregnancy characteristics among congenital syphilis cases after a maternal diagnosis, Uruguay, 2014–2017^a^

Variable	2014 CS = 107, no CS = 300	2015 CS = 88, no CS = 339	2016 CS = 71, no CS = 2 58	2017 CS = 47, no CS = 228
	CS/No CS	CS/No CS	CS/No CS	CS/No CS
Fewer than 5 antenatal checks, n (%)	57/21 (54.3/7.1)	50/35 (56.8/10.4)	32/29 (45.1/11.2)	26/25 (55.3/11.0)
Pregnancy diagnosis (weeks), mean ± SD	14 ± 7/ 12 ± 6	13 ± 8/ 12 ± 6	11 ± 9/ 12 ± 7	12.5 ± 9/ 11.4 ± 6
Pregnancy diagnosis at > 14 weeks, n (%)	50/72 (59.5/28)	33/88 (52.4/26.2)	22/62 (40.7/25)	17/56 (44.7/24.8)
GA at first syphilis test in pregnancy, mean ± SD	–	21 ± 10/ 15 ± 7	15 ± 12/ 14 ± 8	14 ± 11/ 12 ± 6
First syphilis test > 14 weeks, n (%)	30/116 (70/39.3)	42/134 (79/40)	31/112 (69/44)	34/80 (89.5/35.4)
GA at syphilis diagnosis, mean ± SD	24 ± 11/ 17 ± 8	28 ± 9/ 17 ± 8	21 ± 15/ 16 ± 10	20 ± 15/ 16 ± 8
Diagnosis of syphilis at delivery or puerperium, n (%)	64/5 (59.8/1.7)	35/5 (39.8/1.5)	26/2 (36.6/0.8)	17/2 (36.2/0.9)
Correct treatment, n (%)	3/260 (3/96.7)	8/335 (9.1/99.1)	18/236 (26.9/95.1)	4/212 (9.3/99.5)
Treated partners excluding missing data, n (%)	29/115 (58/67.6)	13/144 (25.5/64.3)	17/99 (41.5/65.6)	10/96 (43.5/68.0)

*CS* congenital syphilis, *SD* standard deviation, *GA* gestational age

^a^All denominators exclude cases with missing data

In the last 3 years several variables that characterize sociocultural vulnerability were incorporated into the analysis. This showed that, in exposed children, the main characteristics related with congenital syphilis were public health care, unplanned pregnancy, drug use, history of syphilis, and having previous children with congenital syphilis (Additional file 1: Table S1).

**Fig. 2:**
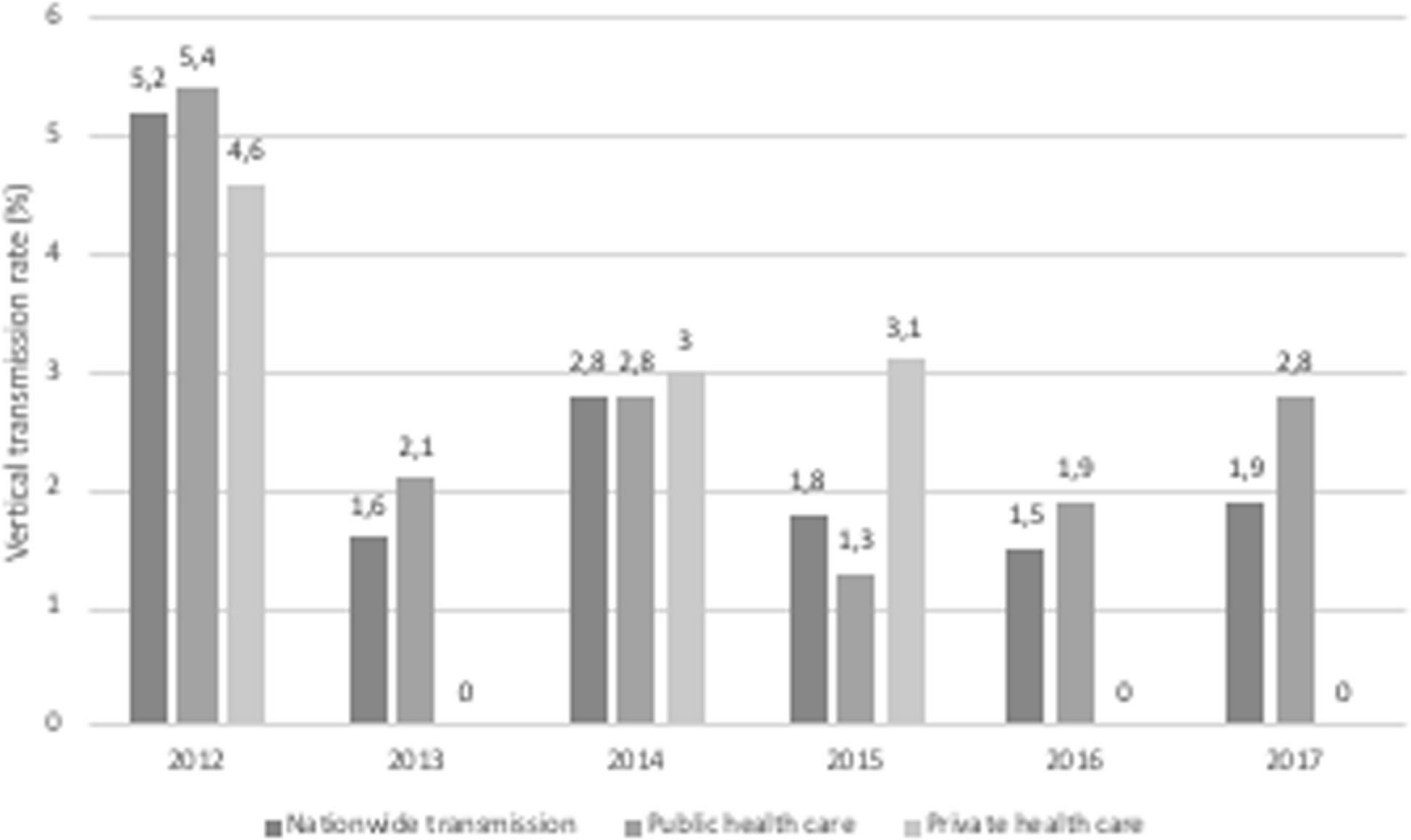
Evolution of vertical HIV transmission in Uruguay according to health care sector, 2012–2017

Regarding mother-to-child transmission (MTCT) of HIV, a decrease was recorded at the national level, from 6.3% in 2012 to 1.9% in 2017, slightly higher than the 2016 rate (1.5%). The rate was below the elimination target in the 2 final years, but in 2017 it was higher in the public health care sector (Fig. 2).

We observed that, in the period of 2012–2016, a lower proportion of women with HIV had an adequate number of antenatal checks (≥ 5 checks), but in 2017 there was an increase of 3.2 percentage points. Early pregnancy diagnosis (≤ 14 weeks) was also found for a lower proportion of women. The increase in HIV testing coverage persists, and remains above 95%, while HIV prevalence has remained stable during this period (data not shown).

The coverage by ART prophylaxis for MTCT was high, as was the coverage by ART, with a constant gestational age at the start of ART. A sustained increase in the proportion of women presenting with undetectable viral load at delivery was also noted (≤ 4 weeks prior to delivery), as well as in the percentage of women who effectively continued ART after delivery. The rate of C-sections among those with indication (data not shown) had a decrease of 6.4 percentage points in 2017.

All the children with HIV were reported in the routine information system. HIV case reviews aimed at detecting system failures or bottlenecks leading to vertical transmission of HIV infection. Additional file 1: Table S2 shows some characteristics of the children with perinatal HIV infection in 2013–2017 according to the case reviews. HIV diagnosis was delayed in the mothers, with one case diagnosed at delivery and two in the puerperium. There were three cases of seroconversion during the pregnancy and one possible late conversion during pregnancy or breastfeeding. In three women, no MTCT prevention strategies were used, and two only received intrapartum AZT. In two out of four women with information on viral load prior to the delivery, cell count was < 1000 copies/mL.

## Discussion

In Uruguay, the rate of congenital syphilis detected by case reviews was higher than that detected with the routine reporting system for all years investigated, showing a persistent underreporting issue. This finding reinforced the need to maintain routinely congenital syphilis case- studies as a surveillance tool. It was also observed that the country has not yet reached the congenital syphilis elimination target. For HIV, however, the EMTCT target was reached in the 2 last years of analysis. The founded congenital syphilis rate is is compatible with the Region of America’s rate of 1.7 cases per 1000 live births in 2015. In 2015, 22 countries and territories in the Region of the Americas reported data compatible with achievement of the goal and targets of HIV MTCT elimination, and 20 reported data compatibles with elimination of MTCT of syphilis [13].

A decline in congenital syphilis was observed for both public and private health care, except for public health care outside the capital metropolitan area. For the private health care sector, the rate in the interior of the country was higher than in the capital, possibly due to different quality of care, but it is already bellow the elimination rate. This suggests the need for a disaggregated evaluation of antenatal care indicators, with separate analysis of capital and non-capital geographical areas to identify possible failures in antenatal care for congenital syphilis prevention. Studies performed in developing countries have reported regional inequalities in antenatal care, resulting in part from difficulties in the access to health care services and low levels of economic development [14–16]. In New York city, in approximately one third of the reported cases of congenital syphilis from 2010 to 2016, the major contributing factor was late initiation of prenatal care, with lack of health care coverage often cited by patients as a barrier to seeking care. The authors concluded that absent or late prenatal care among mothers of infants with congenital syphilis suggests that pregnant women with syphilis might be unaware of available services or face barriers to obtaining prenatal care; this might be particularly applicable for women born outside the United States [17]. Also, in England, cases have been associated with mothers who were socially marginalized and encountered barriers when accessing antenatal care [18]. In Argentina, analyses utilizing data from SIP (2004–2009) also found a higher risk of maternal syphilis among mothers with no antenatal care and in unplanned pregnancies [19].

Among the cases of congenital syphilis in years 2014–2017 we found a higher frequency of congenital syphilis in the presence of late pregnancy diagnosis, fewer than 5 antenatal checks, delayed diagnosis of congenital syphilis at delivery or puerperium, lower rate of correct treatment of congenital syphilis, and untreated partners. The percentage of pregnant women who received antenatal care from skilled professionals was high in Uruguay (> 95%). However, the percentage of women with the first antenatal consultation before 20 gestational weeks ranged from 83.4 to 85.5%. This shows the need to strengthen strategies to increase early antenatal care, especially in vulnerable women, since the performance of tests at the first antenatal check is high in the country. We did not find a higher frequency of congenital syphilis in any maternal age range, as reported by a Brazilian study that found an association with the mother being younger than 20 years [20], but the association of congenital syphilis with low educational attainment, delayed start of antenatal care, and fewer antenatal visits is well established in the literature [20–22].

A regional audit with 42 pregnant women with syphilis in England, found that the national standards were met in the majority of cases with 69% being treated according to national guidance and all cases completing treatment. Locally developed standards on multidisciplinary working and communication were met less well, with particular issues regarding the documentation of pregnancy outcomes in the records and communication between medical specialties being highlighted. The research resulted in a regional good practice guide to address standards not met, reduce adverse outcomes and prevent future cases of congenital syphilis [23].

Sociocultural vulnerability is an important factor in congenital syphilis. We observed a higher frequency of congenital syphilis in the groups of low maternal schooling, unplanned pregnancy, history of syphilis, and having other children with congenital syphilis. The prevalence of absent family planning in Uruguay remained around 33% from 2014 to 2016, showing a possible opportunity for action in the sense of providing better access to contraception, thus preventing unplanned pregnancies that usually lead to worse antenatal care. A significant decline in the fertility rate has been noted in the last biennium, especially among adolescents, following the availability and access to subdermal implants in the first level of care [24].

Preventing congenital syphilis is not technically difficult, however operational difficulties limit the effectiveness of programmes in many settings. A study conducted in Bolivia, Kenya, and South Africa found that early antenatal syphilis screening and management of positive cases were difficult to implement since most women presented for their first antenatal clinic visit after 6 months of pregnancy, the availability of the tests results was variable, and no clinic had a system for tracking RPR-reactive women who did not return for their results. The authors also mention a need to improve training and work conditions for the health-care providers and drugs availability [25].

In relation to congenital syphilis, several prevention strategies could be implemented at the primary care level, including encouraging women to attend antenatal care before the 4th month of pregnancy, providing point-of-care testing so that results are available immediately and women who test positive can be treated, implementing presumptive treatment of sexual partners of women who test positive, adding a second test later in pregnancy so that incident cases can be managed, and improving the quality of syphilis care during pregnancy, delivery, and the neonatal period [25].

Regarding MTCT of HIV, reporting via the routine information system was 100%. Nevertheless, the case reviews focusing on infected newborns are still useful as an opportunity to identify points of system failure, including delayed pregnancy diagnosis, delay in the definition of maternal infection status even in women who began antenatal care after 14 pregnancy weeks, and lack of treatment adherence, among others. Regarding HIV, despite an early pregnancy diagnosis (on average 12 weeks), gestational age at HIV diagnosis has remained at 18–19 weeks. This might be explained by the use of immunoenzymatic assays as screening technique for HIV at the first routine testing in pregnancy, with communication of results only after a confirmation is obtained by Western-Blot, a deep-rooted practice among technicians, which persists despite the changes in diagnostic protocols. A decrease was noted in the diagnosis performed at delivery or immediate puerperium (rapid test).

Our study has strengths as the high coverage of the audits and the fact that the audits also take in account all positive mothers, not only positive neonates, increasing their capacity to find cases. These also allow the identification of weakness in the health care process and to perform feedback for the improvement process. As limitations we consider that, as a retrospective analysis, the identification of positive women occurs during delivery and post-partum. The ideal scenario was that a positive test in a pregnant woman, both for syphilis and HIV, would trigger an alert in the system allowing supervision of medical conducts and the adequate follow -up, helping to avoid cases of maternal to child transmission.

## Conclusions

Case reviews of congenital syphilis in Uruguay have produced knowledge regarding the real number of cases and allowed an assessment of the status of EMTCT in the country. The results suggest that rates must be adjusted and provided an opportunity to improve the reliability of surveillance data. Also, there is a need to maintain routinely congenital syphilis case- studies as surveillance tool. For HIV, the case reviews showed the need to better inform health care professionals regarding diagnostic protocols. In relation to congenital syphilis, several prevention strategies could be implemented including encouraging women to attend antenatal care before the 4th month of pregnancy thus increasing the odds of pregnant women and their partners being diagnosed and treated earlier and improving health care workers training in congenital syphilis diagnostic and management.

## Supplementary information

**Additional file 1: Table S1.** Sociocultural characteristics among congenital syphilis cases after a maternal diagnosis, Uruguay, 2015–2017. **Table S2.** Analysis of cases resulting in vertical HIV transmission, Uruguay, 2013–2017. (DOCX 34 kb)

## Data Availability

The original data source for this article is available at https://www.gub.uy/ministerio-salud-publica/tematica/itsvih-sida?page=1.

## Abbreviations

ART: Antiretroviral therapy
AZT: Azidothymidine
CS: Congenital syphilis
DEVISA: Bureau for Epidemiological Surveillance
EMTCT: Elimination of mother-to-child transmission
GA: Gestational age
HDI: Human development index
HIV: Human Immunodeficiency Virus
LBC: Electronic live birth certificate
MDGs: Millennium Development Goals
MTCT: Mother-to-child transmission
PAHO: Pan American Health Organization
RPR: Rapid Plasma Reagin
SD: Standard deviation
SIP: Perinatal Information System (*Sistema Informático Perinatal)*
SNIS: Integrated National Health Care System (*Sistema Nacional Integrado de Salud*) SNS National Health Insurance (*Seguro Nacional de Salud*)
STI: Sexually transmitted infections
VDRL: Venereal Disease Research Laboratory
WHO: World Health Organization
