# Supplementary material for: Lessons about the reliability of congenital syphilis and vertical HIV transmission data learned from case reviews in Uruguay: a cross-sectional study
Source: BMC Pregnancy Childbirth. 2019 Nov 4;19:400. doi: 10.1186/s12884-019-2516-z (PMC6827247; doi:10.1186/s12884-019-2516-z)
Supplement: Supplementary file 1 — Additional file 1: Table S1. Sociocultural characteristics among congenital syphilis cases after a maternal diagnosis, Uruguay, 2015–2017. Table S2. Analysis of cases resulting in vertical HIV transmission, Uruguay, 2013–2017. (DOCX 34 kb) [file 12884_2019_2516_MOESM1_ESM.docx]

**SUPPLEMENTARY MATERIAL**

**Table S1** Sociocultural characteristics among congenital syphilis cases after a maternal diagnosis, Uruguay, 2015-2017

| **Variable** | **2015**  **(CS=88, no CS=339)** | | **2016**  **(CS=71, no CS=2 58)** | **2017**  **(CS=47, no CS=228)** |  |
| --- | --- | --- | --- | --- | --- |
|  | **CS/No CS** | | **CS/No CS** | **CS/No CS** | |
| Public/private health care, n (%) | 71 (308)/17 (119)  (23.0/14.3) | | 61(308)/10 (119)  (23.0/15.6) | 42(211)/5(64)  (19.9/78) | |
| Age <19 years, n (%) | 11/45  (12.5/13.3) | | 8/32  (11.3/12.5) | 5/16  (10.6/7.0) | |
| Ethnic minority, n (%) | 14/37  (17.3/13.4) | | 11/28  (17/11.5) | 10/36  (23.3/16.4) | |
| Maximum educational attainment, n (%) |  |  |  |  |  |
| Illiterate | 2/0 (2.2/0) | | 0/1 (0/4) | 0/0 | |
| Incomplete elementary | 7/23 (9.8/8.4) | | 3/16 (4.9/6.8) | 3/7 (7.0/3.2) | |
| Complete elementary | 29/90 (38/32.7) | | 27/68 (44.3/28.8) | 15/60 (34.9/27.8) | |
| Incomplete high school | 36/141 (43.5/51.3) | | 28/130 (46/63.6) | 21/129 (48.8/59.7) | |
| Complete high school | 3/14 (3.3/5.1) | | 1/19 (1.6/8) | 1/13 (2.3/6.0) | |
| University level | 2/7 (3.3/2.5) | | 2/2 (3.3/0.8) | 3/7 (7.0/3.2) | |
| Having a partner, n (%) | 51/205  (67.1/75.9) | | 35/147  (56.4/61.8) | 25/131  (59.5/61.5) | |
| Planned pregnancy, n (%) | 24/131  (30.4/48.3) | | 14/87  (22.6/36.9) | 13/96  (31.0/44.4) | |
| Drug use, n (%) | 14/14  (19.2/5.4) | | 5/9  (9.3/4.1) | 10/21  (37.0/10.9) | |
| Alcohol use, n (%) | 7/11  (9.7/4.2) | | 5/13  (9.1/5.8) | 2/16  (7.4/6.3) | |
| Violence, n (%) | 5/4  (7/1.5) | | 5/9  (9.3/4.1) | 0/4  (0/2.1) | |
| Previous syphilis, n (%) | 15/34  (20.5/11.7) | | 14/25  (23.7/10.7) | 11/22  (24.4/10.1) | |
| Children with CS, n (%) | 6/2  (40/5.9) | | 5/4  (55.6/25) | 3/3  (7.3/1.4) | |

CS, congenital syphilis.

**Table S2** Analysis of cases resulting in vertical HIV transmission, Uruguay, 2013-2017

| **Variable** | **2013** | | **2014** | | | | **2015** | | **2016** | | **2017** |  |
| --- | --- | --- | --- | --- | --- | --- | --- | --- | --- | --- | --- | --- |
|  | **Case 1** | **Case 2** | **Case 1** | **Case 2** | **Case 3** | **Case 4** | **Case 1** | **Case 2** | **Case 1** | **Case 2** | **Case 1** | **Case 2** |
| Maternal age (years) | 22 | 40 | 38 | 31 | 23 | 17 | 34 | 26 | 19 | 19 | 27 | 34 |
| ≥ 5 antenatal control visits | No | Yes | No | No | Yes | Yes | Yes | Yes | No | No | Yes | Yes |
| Gestational age at first check (weeks) | 24 | 10 | UN | SD | 17 | 12 | 19 | 17 | 24 | 12 | 10 | 15 |
| HIV diagnosis | AD | Puerperium | UN | SD | 17 weeks | 32 weeks | 24 weeks | 32 weeks | 24 weeks | UN | Puerperium | Previous |
| GA at first test during pregnancy (weeks) | 28 | 12 | UN | NC | 17 | 12 | 19 | 17 | 24 | UN | - | - |
| ART | No | No | No | Yes | Yes | No | Yes | Yes | Yes | Yes | No | Yes |
| GA at start of ART | - | - | - | 32 | 28 | UN | 26 | 37 | 34 | 12 | - | 29 |
| AZT | Yes | No | Yes | Yes | Yes | No | Yes | Yes | Yes | Yes | No | Yes |
| Single dose nevirapine | No | No | No | No | No | No | No | No | No | No | No | No |
| C-section | Yes | Yes | Yes | Yes | Yes | No | Yes | Yes | Yes | Yes | No | Yes |
| Viral load at delivery | UN | UN | UN | 3923 cop/mL | UN | UN | 78 cop/mL | UN | 1460 cop/mL | 44 cop/mL | - | UN |
| NB prophylaxis | AZT + NVP | No | AZT | AZT + NVP | AZT + NVP | AZT | AZT | AZT | AZT + NVP | AZT | No | AZT/NVP |
| Interruption of breastfeeding | Yes | No, initially | Yes | Yes | Yes | Yes | Yes | Yes | Yes | Yes | No | Yes |

AD, at delivery; ART, antiretroviral therapy; GA, gestational age; NB, newborn; NVP, nevirapine; UN, Unknown; VL, viral load.
